# Osteoporotic vertebral fractures and subsequent fractures: risk factors from a retrospective observational study of patients with osteoporosis

**DOI:** 10.3389/fmolb.2025.1558052

**Published:** 2025-03-19

**Authors:** Mingxing Fan, Ran Lu, Jiayuan Wu, Jie Huang, Yanming Fang

**Affiliations:** ^1^ Department of Spine Surgery, Beijing Jishuitan Hospital, Capital Medical University, Beijing, China; ^2^ Department of Endocrinology and Metabolism, Peking University Third Hospital, Beijing, China

**Keywords:** subsequent fracture, osteoporosis, risk factor (RF), retrospective observational study, bone mineral denisty

## Abstract

**Purpose:**

Osteoporosis is a progressive, systemic, skeletal disorder characterized by increased bone fragility and susceptibility to fracture. Prior fractures are a strong predictor of subsequent fractures, but it is essential to identify further clinical and demographic characteristics of patients with osteoporosis that are associated with subsequent fracture risk.

**Methods:**

In this retrospective observational cohort study, male and female patients over the age of 55 years with osteoporosis who experienced vertebral fractures between 2019 and 2021 were included. All patients’ basic clinical data, serum biochemical and bone turnover markers, bone mineral density, and other indicators were recorded uniformly. The incidence of subsequent fractures during the two-year follow-up period was analyzed. Independent risk factors for subsequent fractures were identified by binary logistic regression analysis.

**Results:**

A total of 1,096 patients were included. Of these, 311 (28.4%) patients suffered a subsequent fracture during the two-year follow-up period. The incidences of subsequent fracture sites were 18.4% vertebral, 14.2% forearm/wrist/hand, and 9.9% hip/femur. Compared with the non-subsequent fracture group (non-SFG), binary logistic regression analysis showed that body mass index (BMI) (OR [95% CI] 0.825 [0.720–0.945]; P = 0.006), femoral neck bone mineral density (BMD) T-score (OR [95% CI] 0.067 [0.012–0.385]; P = 0.002), and C-terminal telopeptide of type 1 collagen (CTX) levels (OR [95% CI] 6.089 [1.735–21.375]; P = 0.005) were independent risk factors associated with subsequent fractures.

**Conclusion:**

Patients with osteoporosis and previous vertebral fractures are at a higher risk of further fractures at a two-year follow-up period. BMI, femoral neck BMD T-score, and CTX levels were independent risk factors for refracture. Integrating BMI, femoral neck BMD, and CTX levels into an individualized care plan for patients with osteoporotic vertebral fractures may help prevent subsequent fractures in high-risk populations.

## Background

Osteoporotic fractures can lead to a range of debilitating symptoms, including lower back pain, vertebral collapse, and restricted mobility, severely impacting patients’ quality of life. They are a major cause of disability, loss of independence, and reduced quality of life (Beall et al., 2018; Yang et al., 2020). Existing vertebral fractures significantly increase the likelihood of subsequent fractures in patients with osteoporosis, particularly at the site of the hip/femur, vertebra, and forearm/wrist/hand (Hadji et al., 2021). Notably, 10%–29.8% of individuals experience a subsequent fracture within 1–2 years following an initial fracture (Balasubramanian et al., 2019; Lee et al., 2019). Among various types of osteoporotic fractures, vertebral fractures are the primary site carrying the highest cumulative risk for subsequent fractures (Balasubramanian et al., 2019). Given this significant burden, it is crucial to investigate the risk factors associated with recurrent fractures in patients with initial vertebral fractures and to develop targeted preventive and therapeutic interventions to address these risks. In the present study, we evaluated the imminent risk of subsequent fractures in patients with osteoporosis and initial vertebral fractures to identify clinical and demographic factors that are independently associated with the risk of subsequent fractures.

## Methods

This study was a retrospective observational cohort study conducted at a single center. Male and female patients aged over 55 years with osteoporosis who experienced at least one vertebral fracture between 2019 and 2021 were included. This study was approved by the Ethics Committee, with the approval number K2023-085-00. The inclusion criteria were as follows: patients aged over 55 years; vertebral L1–L4 bone mineral density (BMD) T-score < −2.5 standard deviation (SD) as measured by quantitative computed tomography (QCT); diagnosis of at least one vertebral fracture; and a signed informed consent form. The exclusion criteria were fractures caused by high-energy trauma (e.g., motor vehicle accidents, falls from height, or other high-energy impacts) and pathological fractures or other metabolic bone diseases secondary to drug use, steroid therapy, or other factors. All patients were advised to receive regular anti-osteoporosis treatments after their first fracture, with medication guidance provided by physicians (calcium and vitamin D supplementation and anti-osteoporosis therapy with denosumab or bisphosphonates). Basic clinical data, serum biochemical markers, bone metabolic markers, vertebral L1–L4 BMD, femoral neck BMD T-score, and other relevant parameters were uniformly recorded for all patients. The incidence and timing of subsequent fractures, including hip/femur, vertebral, and forearm/wrist/hand fractures, were analyzed during a two-year follow-up. Independent risk factors for subsequent fractures were identified through binary logistic regression analysis.

### Basic clinical data

General clinical information, including age, height, weight, body mass index (BMI), and combined disease, such as diabetes, rheumatic disease, chronic obstructive pulmonary disease, cardio-cerebrovascular diseases, chronic gastric diseases, and chronic liver or kidney diseases, was recorded.

### Vertebral L1–L4 BMD and femoral neck BMD T-score

Vertebral L1–L4 BMD was measured using QCT, and the average value of L1–L4 was calculated. T-scores and Z-scores were derived accordingly (IACoR, 2008). Femoral neck BMD was measured using dual-energy X-ray absorptiometry (DEXA), and the corresponding T-scores were recorded.

### Serum biochemical and bone turnover markers

Early morning fasting venous blood samples were collected from all enrolled patients. The primary markers measured included serum calcium (Ca), serum phosphorus (P), serum parathyroid hormone (PTH), serum 25-hydroxyvitamin D (25-OHD), serum bone Gla protein (BGP), serum C-terminal telopeptide of type 1 collagen (CTX), and procollagen type 1 N-terminal propeptide (PINP).

### Statistical analysis

All data analyses were performed using IBM SPSS (version 29.0 IBM Co., Armonk, NY, United States). Descriptive statistics were calculated to summarize patients’ baseline characteristics and the incidence of the index and subsequent fractures. Absolute counts and percentages are presented for categorical variables. The chi-square test and Fisher’s exact test were used to evaluate differences between categorical variables. Univariate analyses were conducted to explore potential risk factors for subsequent fractures. Binary logistic regression analysis was performed to identify independent risk factors for subsequent fractures. A p-value of less than 0.05 was considered statistically significant.

## Results

A total of 1,096 patients were included in the study. Among them, 311 patients (28.4%) experienced a subsequent fracture during the two-year follow-up period. The incidences of subsequent fracture sites were 18.4% vertebral, 14.2% forearm/wrist/hand, and 9.9% hip/femur.

Compared with the non-subsequent fracture group (non-SFG), the subsequent fracture group (SFG) had a significantly lower BMI (P < 0.01). Although the height and weight in the SFG were lower than those in the non-SFG, the difference was not statistically significant (P > 0.05). There was no significant difference in the incidence of comorbidities between the two groups (Table 1).

**TABLE 1:** Patient demographics and comorbidities in SFG and non-SFG.

Parameter	SFG (n = 311)	Non-SFG (n = 785)	P-value
Age (years)	75.00 (66.00, 79.00)	73.00 (68.00, 80.00)	0.343
Height (m)	1.60 (1.57, 1.66)	1.60 (1.55, 1.66)	0.061
Weight (kg)	60.50 (55.00, 70.00)	62.00 (55.00, 70.00)	0.081
BMI (kg/m^2^)	23.58 (21.48, 25.80)	24.65 (22.22, 26.67)	0.002*
Male (%)	19.0	20.9	0.477
Smoking History (%)	6.4	5.9	0.746
Alcohol History (%)	1.6	3.0	0.204
Hypertension (%)	49.8	53.2	0.314
Diabetes (%)	13.5	14.9	0.552
Rheumatic Disease (%)	1.3	1.9	0.475
Chronic obstructive pulmonary disease, (%)	1.3	1.0	0.702
Chronic gastric diseases (%)	2.6	3.6	0.405
Chronic liver or kidney diseases (%)	1.6	1.5	0.924

Compared with the non-SFG, the SFG exhibited significantly lower vertebral L1–L4 BMD (P < 0.01), T-scores (P < 0.01), and Z-scores (P < 0.01), as well as reduced femoral neck BMD T-score (P < 0.01; Table 2). Regarding serum biochemical and bone metabolic markers, significant differences were observed in 25-OHD (P = 0.005) and CTX levels (P = 0.037).

**TABLE 2:** BMD, serum biochemical and bone turnover markers in SFG and non-SFG.

Parameter	SFG (n = 302)	Non-SFG (n = 800)	P-value
Femoral neck BMD T-score	−5.31 (−5.67, −4.83)	−4.38 (−4.96, −3.58)	<0.001*
Vertebral L1–L4 BMD (mg/cm^3^)	34.8 (25.00, 46.90)	63.30 (39.85, 78.90)	<0.001*
Vertebral L1–L4 BMD Z-score	−1.50 (−2.19, −0.92)	−1.23 (−1.59, −0.52)	0.005*
Vertebral L1–L4 BMD T-score	−4.92 (−5.51–4.50)	−4.04 (−4.71, −3.44)	<0.001*
PINP (ng/mL)	66.58 (41.65, 88.43)	66.36 (44.06, 85.30)	0.926
25-OHD (ng/mL)	14.34 (7.95, 18.32)	14.97 (9.78, 21.33)	0.005*
Calcium (Ca, mmol/L)	2.23 (2.21, 2.29)	2.26 (2.17, 2.39)	0.142
Phosphorus (P, mmol/L)	1.01 (0.99, 1.21)	1.07 (1.01, 1.15)	0.084
PTH (pg/mL)	54.30 (42.20, 54.80)	52.20 (36.40, 66.10)	0.972
BGP (ng/mL)	17.80 (7.80, 26.56)	17.30 (13.04, 21.11)	0.147
CTX (ng/mL)	0.81 (0.55, 1.02)	0.62 (0.41, 0.76)	0.037*

We performed binary logistic regression analysis on the identified factors associated with subsequent fractures. Compared with the non-SFG group, binary logistic regression analysis revealed that BMI (OR = 0.825, 95% CI 0.720–0.945) and femoral neck BMD measured by DEXA (OR = 0.067, 95% CI 0.012–0.385) were protective factors, and elevated serum CTX levels (OR = 6.089, 95% CI 1.735–21.375) was a risk factor for subsequent fractures within 2 years in patients with osteoporosis (Table 3). The accuracy of the model was 87.4%. AUC-ROC (area under curve-receiver operating characteristic curve) was 0.910 (95% CI: 0.858–0.962), with a maximum Youden index of 0.705. The corresponding sensitivity was 0.765, with a specificity of 0.940.

**TABLE 3:** Multivariate analysis for subsequent fractures, conducted through binary logistic regression.

Risk factor	OR	95% CI	P
BMI (kg/m^2^)	0.825	0.720–0.945	0.006
Femoral neck BMD T-score	0.067	0.012–0.385	0.002
Vertebral L1–L4 BMD (mg/cm^3^)	0.952	0.892–1.016	0.138
Vertebral L1–L4 BMD Z-score	1.630	0.661–4.021	0.289
Vertebral L1–L4 BMD T-score	2.770	0.408–18.799	0.297
25-OHD (ng/mL)	1.022	0.960–1.088	0.493
CTX (ng/mL)	6.089	1.735–21.375	0.005

## Discussion

Vertebral fractures indicate a high risk of subsequent fracture in patients with osteoporosis (Hadji et al., 2021; Ross et al., 1991; Klotzbuecher et al., 2000; Lindsay et al., 2001; Wong et al., 2022). Recurrent fractures in patients with osteoporosis with initial vertebral fractures result in a significant clinical and societal burden (Kocijan et al., 2024; Betancur et al., 2024; Ju and Liu, 2023). This study provides valuable insights into the imminent risk of subsequent fractures among patients with osteoporosis who have suffered initial vertebral fractures, highlighting key demographic, radiological, and biochemical factors associated with subsequent fractures. The findings have important implications for early intervention and management of subsequent fractures associated with osteoporosis.

The study revealed that approximately 28.4% of patients experienced a subsequent fracture within the two-year follow-up. Notably, vertebral fractures accounted for the highest proportion (18.4%) of subsequent fractures. The rate of subsequent fracture in this study was similar to the 25.5% incidence of subsequent fractures after prior fractures reported by Balasubramanian et al. (2019) at two-year follow-up, which was higher than the overall 13.4% incidence reported in the meta-analysis by Dai et al. (2022) after osteoporotic vertebral compression fracture. The reasons for this include (1) the incidence of refracture was affected by the duration of follow-up, which ranged from 4 to 52.5 months in the meta-analysis (Dai et al., 2022); (2) different populations had different levels of adherence to osteoporosis treatments, which affected the treatment of osteoporosis. Betancur et al. (2024) reported that 70.5% of patients in Colombia suffered a refracture 7 months after osteoporosis fracture due to a lack of standardized treatment. The higher rate of refracture in this study emphasizes the urgent need for targeted prevention strategies for this high-risk population. Identifying risk factors for subsequent fractures and developing individualized treatment plans play a key role in addressing the challenge of subsequent fractures in patients with osteoporosis.

BMI was significantly associated with a decreased risk of subsequent fractures (OR = 0.825, P = 0.006). Several studies (Ju and Liu, 2023; Yang et al., 2016; Wang et al., 2014) have concluded that BMI is not significantly associated with refracture in patients with osteoporosis. However, Özmen et al. (2024) suggested that BMI (OR = 0.93, P < 0.001) is a protective factor for osteoporosis. Dovjak et al. (2024) concluded that lower BMI (hazard ratio, HR = 0.925, P = 0.009) is associated with a higher rate of further fractures in patients with fragility fractures. Cheng et al. (2024) also found that BMI was a protective factor (OR = 0.863, P = 0.003) for subsequent fractures in patients with osteoporotic vertebral fractures. Lower BMI may be related to less mechanical loading on bones, reduced estrogen secreted from androgen receptors in adipose tissue (Özmen et al., 2024), and a higher risk of malnutrition and the development of sarcopenia (Dovjak et al., 2024), together increasing the risk of subsequent fractures. This conclusion needs to be validated in prospective studies.

This study found that patients with higher femoral neck BMD T-score had a lower risk of subsequent fracture (OR = 0.067, P = 0.002). Femoral neck BMD as a risk factor for refracture in patients with osteoporosis fracture has been validated in several studies (Arceo-Mendoza and Camacho, 2021; Center et al., 2007; Mazzantini et al., 2020). However, there was no significant correlation between vertebral L1–L4 BMD and refracture (OR = 0.952, P = 0.138). Ju and Liu (2023) found that vertebral L1–L4 BMD (OR = 0.78, P = 0.011) could be a predictor of spinal refracture after osteoporotic vertebral compression fractures. Cheng et al. (2024) found the Hounsfield unit (HU) value (OR = 0.976, P < 0.001) was an independent risk factor of adjacent vertebral fracture following osteoporotic vertebral compression fracture (OVCF). Because the refracture statistics in this study included vertebral fractures and fractures at other sites (hip/femur and forearm/wrist/hand fractures) and the uncertainty in the prediction of non-vertebral fractures by vertebral L1–L4 BMD, this may have reduced the correlation between them (Tan et al., 2022). This study reinforces the utility of femoral neck BMD T-score measurements in stratifying subsequent fracture risk in patients with osteoporosis.

Among the serum biochemical and bone metabolic markers analyzed, elevated CTX levels emerged as an independent risk factor for subsequent fractures (OR = 6.089, P = 0.005). CTX reflects bone resorption activity and is a recognized marker for monitoring osteoporosis treatment response (Hlaing and Compston, 2014; Vasikaran et al., 2011). Elevated CTX levels in the SFG suggest increased bone resorption, which weakens bone microarchitecture and increases the risk of subsequent fractures. A meta-analysis by Tian et al. (2019) showed that CTX was positively associated with fracture risk (crude gradient of risk, 1.16; adjusted gradient of risk, 1.20). Ivaska et al. (2022) found CTX was predictive of fractures over 1–3 years (hazard ratios ranging from 1.28 to 2.28 per SD). However, Zhu et al. (2022) concluded that CTX was not associated with refracture in patients with osteoporosis. CTX may be influenced by circadian variation, food intake, glucose status, and renal failure (Hlaing and Compston, 2014), and the reference interval of CTX is very large (100–700 ng/L) (Wu et al., 2021), so CTX may vary between different osteoporotic fracture populations. Our study clarifies the significance of CTX in predicting the risk of refracture in patients with OVCF, and changes in CTX should be considered when assessing the risk of refracture in patients with OVCF.

In this study, a predictive model for refracture in patients with OVCF at two-year follow-up was constructed using BMI, femoral neck BMD T-score, and CTX, and the model had a good predictive effect on patient prognosis (AUROC, 0.910). Different studies have identified different risk factors for refracture in patients with osteoporosis. For example, Hadji et al. (2021) identified the initial fracture site, age, antiepileptic medication use, and heart failure risk factors as risk factors for refracture in patients with osteoporosis, whereas Zhu et al. (2022) found that the risk factors of refracture in patients with osteoporosis were advanced age, overweight, and decreased BMD. These differences may be related to the demographic information, medical conditions, and adherence of the included population, as well as the setting of the study’s observational and outcome metrics. Future multicenter prospective studies will better validate the risk of refracture after initial fracture in patients with osteoporosis. The risk factors identified in this study may assist physicians in identifying patients at high risk of refracture and in adjusting interventions accordingly. Promoting improved nutritional status, anti-osteoporosis treatment, and optimization of anti-osteoporosis programs through monitoring of bone turnover markers is essential to prevent refracture.

This study has several limitations. First, this study is a single-center retrospective study, and the results need to be verified by prospective multicenter studies. Second, the follow-up time of this study was 2 years after the initial fracture in patients with osteoporosis, which is the period with the highest risk of refracture (Johansson et al., 2017; Roux and Briot, 2017). However, the patients were still at risk of refracture after 2 years, and we will follow up with the patients further. Third, treatment regimens of patients with osteoporosis after vertebral fracture and adherence to anti-osteoporosis treatment are closely related to the patients’ prognosis, which we will investigate in future studies.

## Conclusion

This study confirms that patients with osteoporosis with initial vertebral fractures are at higher risk of refracture at 2-year follow-up and identifies BMI, femoral neck BMD T-score, and CTX levels as independent risk factors for subsequent fractures. Therefore, integrating BMI, femoral neck BMD, and CTX levels into an individualized care plan for patients with osteoporotic vertebral fractures can help physicians develop individualized prevention and treatment strategies for high-risk populations, enhance the prevention of subsequent fractures, and improve the quality of life of patients with osteoporosis.

## Data Availability

The original contributions presented in the study are included in the article/supplementary material, further inquiries can be directed to the corresponding author.
